# Unusual Complication Attending Septicæmia Following Parturition; Autopsy

**Published:** 1895-09

**Authors:** M. O’M. Knott

**Affiliations:** House Surgeon, Hospital of New York College of Veterinary Surgeons


### UNUSUAL COMPLICATION ATTENDING SEPTICÆMIA FOLLOWING PARTURITION; AUTOPSY.

BY M. O'M. KNOTT, V.S.,
HOUSE SURGEON, HOSPITAL OF NEW YORK COLLEGE OF VETERINARY SURGEONS.

A GREAT Dane bitch, apparently in good health, whelped on May 7, dropping a litter of twelve pups. Three or four days later the owner noticed that she had become suddenly ill and was giving very little milk; diarrhœa and loss of flesh were also notable symptoms. General prostration followed, the pups began to die, and finally only three were left. Fourteen days after delivery the owner thought she had more pups, because her abdomen had become large again; the swelling gradually increased, and the animal became more emaciated and weaker. On May 30 she was sent to the hospital. Examination on entrance showed a well-developed emaciated dog, weighing about seventy pounds; temperature normal; pulse 60, hard and full; the extremities of the limbs slightly swollen; the visible mucous membranes dull yellow; the abdomen greatly swollen and fluctuation evident; marked dyspnœa and diarrhœa, with offensive odor. On the 31st I tapped her in the posterior part of the abdomen, a little to the left of the median line, and drew off about three quarts of almost clear fluid, with a slight yellowish tinge. The patient was much relieved, and the next day her appetite was better and she appeared brighter. The improvement did not last many days, however; she gradually became weaker and more emaciated, vomiting dark matter of an offensive odor, and died on June 11.

The treatment consisted of a diet of boiled milk, eggs, and whiskey; large doses of potassium iodide, iron and quinine citrate, and small doses of strophanthus by mouth, with enemata of oil, opium, and carbolic acid.

*Autopsy.* On making an incision into the abdomen considerable fluid like that obtained by tapping was found. The peritoneum, both visceral and parietal, was injected and had lost its gloss. The intestines throughout were inflamed, ulcerated in parts, and in one spot were gangrenous. Both horns of the uterus were greatly swollen and ulcerated, and the internal lining was covered with a muco-purulent coating. The spleen was greatly enlarged, being 12½ inches long and its weight 270 grammes. On section numerous hemorrhages were evident. The liver showed a general yellowish tinge (icterus). The bloodvessels of the stomach and mesentery were injected. The lungs were œdematous. The left ventricle of the heart was much dilated, and a thrombus was found adhering to the mitral valve.

The microscopical examination of the organs by Dr. G. P. Biggs is appended.

*Heart.* The muscle-fibres were found to be very granular and contained a few minute fat-globules.

*Kidneys.* The lesions found in these organs were those of an acute diffuse nephritis of moderate severity; the epithelial cells of the tubules were in various stages of degeneration, some being completely necrosed, some containing large fat-globules, and some simply swollen and granular. The lumena of some of the tubules were filled with cast-off cells and coagulated albuminous material. These changes were most advanced in the convoluted tubules; a few casts were found in the straight tubules. There were numerous areas of small round-cells in the stroma between the tubules, and the organs were congested throughout. A small amount of albuminous exudate was seen between the glomeruli and their capsules.

*Liver.* The liver showed marked passive congestion and parenchymatous degeneration.

*Intestines.* The mucous membrane was swollen and the cells were so degenerated that they would not take nuclear staining. A moderate number of small round-cells were found in the deeper parts of the mucosa and the more superficial parts of the submucosa. Similar cells were seen in the external muscular layer and in the connective-tissue between the external and internal muscular layers.

*Uterus.* The mucous membrane varied much in thickness, but presented everywhere the same character of lesion. The vessels were engorged with blood and the tissues in places the seat of hemorrhages. Small round-cells were abundant between the few remaining tubules of the endometrium. That the entire thickness of the wall was involved by the inflammatory process was shown by the presence of small round-cells in large numbers in the submucous and muscular layers.

Cultures from the spleen remained sterile, showing pretty conclusively that the germs producing the endometritis and peritonitis did not invade the system at large, the lesions of the other organs being due to toxic products manufactured at the site of infection.

The anatomical diagnosis is, therefore, suppurative metritis and endometritis, acute sero-fibrinous peritonitis, ulcerative gastro-enteritis, acute hyperplasia of the spleen, acute diffuse nephritis, parenchymatous hepatitis, œdema of lungs, parenchymatous degeneration of the heart, and thrombus of the mitral valve.

